# Correlation of Two-Dimensional Echocardiography Parameters With Six-Minute Walk Test, Oxygen Saturation, and Spirometry in Interstitial Lung Disease

**DOI:** 10.7759/cureus.91875

**Published:** 2025-09-09

**Authors:** Govind K Kumar, Parinita Suresh, Aleena M Mathew

**Affiliations:** 1 Department of Respiratory Medicine, Rajarajeswari Medical College and Hospital, Bangalore, IND; 2 Department of Pulmonary Medicine, Rajarajeswari Medical College and Hospital, Bangalore, IND

**Keywords:** echocardiography, interstitial lung disease, oxygen saturation, pulmonary hypertension, six-minute walk test, tapse

## Abstract

Background and aim: Interstitial lung disease (ILD) is often complicated by pulmonary hypertension (PH), a combination that strains the right side of the heart and worsens outcomes. Two bedside ultrasound measurements, tricuspid annular plane systolic excursion (TAPSE) and pulmonary artery systolic pressure (PASP), have emerged as convenient ways to gauge right ventricular function and pulmonary vascular load. We set out to determine how well TAPSE and PASP reflect everyday exercise capacity (six-minute walk distance, 6MWD), oxygen saturation, and routine spirometric indices in adults with ILD, in hopes of identifying simple, clinically useful prognostic markers.

Methods: During a three-month prospective study at Rajarajeswari Medical College & Hospital, Bangalore, India, we enrolled 40 adults who met ATS/ERS criteria for ILD. Each participant underwent transthoracic two-dimensional echocardiography, spirometry, and a six-minute walk test. Patients were grouped according to their TAPSE and PASP results, and these groups were compared on exercise performance and lung function metrics. Statistical analysis involved t-tests, one-way ANOVA, Pearson correlation, and Bonferroni-adjusted post hoc tests.

Results: Mildly reduced TAPSE was seen in 50% of the cohort, while 57.5% had elevated PASP. Participants with normal TAPSE and PASP walked farther, maintained higher pre- and post-exercise oxygen saturation, and demonstrated superior forced vital capacity (FVC) and forced expiratory volume in one second (FEV₁). Both TAPSE and PASP correlated strongly and significantly (p<0.001) with all functional measures except the FEV₁/FVC ratio.

Conclusion: TAPSE and PASP closely mirror exercise tolerance, exertional desaturation, and spirometric performance in ILD. Exercise tests such as the six-minute walk test can provide practical prognostic insight in the assessment of ILD.

Clinical implications: Bedside echocardiography using TAPSE and PASP can flag early right ventricular dysfunction and incipient PH, guiding timely intervention. The 6MWD provides an approximate estimate of disease progression even in resource-constrained settings. Thus, the 6MWD may serve as an effective alternative for early detection of complications such as PH where advanced resources are unavailable.

## Introduction

Interstitial lung diseases (ILDs) represent a broad, heterogeneous collection of parenchymal disorders that involve the pulmonary interstitium together with the alveolar epithelium, capillary endothelium, and perivascular and perilymphatic structures. Although diverse in etiology, these conditions share a common tendency to provoke inflammation and subsequent fibrosis of the lung parenchyma, leading to architectural distortion, impaired gas exchange, progressive hypoxemia, and eventually respiratory failure. Despite continued advances in imaging modalities, histopathological techniques, and classification systems, ILD remains clinically challenging because of its highly variable presentation and unpredictable trajectory. The spectrum encompasses idiopathic pulmonary fibrosis (IPF), nonspecific interstitial pneumonia (NSIP), connective tissue disease-related ILDs, hypersensitivity pneumonitis, sarcoidosis, and several other entities [1,2].

One of the gravest complications of ILD is the development of pulmonary hypertension (PH), defined as a resting mean pulmonary arterial pressure (mPAP) ≥20 mmHg confirmed by right heart catheterization, the gold-standard diagnostic test [3]. The reported prevalence of PH in ILD varies widely (≈15-85%) owing to differences in ILD subtype, disease severity, diagnostic criteria, and study populations [4]. When present, PH heralds an exceptionally poor prognosis and is linked to reduced exercise capacity, diminished health-related quality of life, right-sided heart failure, and increased mortality [5].

While right heart catheterization offers a direct measurement of pulmonary hemodynamics, its invasive nature, cost, and limited availability restrict its utility in routine clinical practice, especially in low-resource settings [6]. Thus, transthoracic two-dimensional echocardiography has become a cornerstone non-invasive tool for the initial evaluation of suspected PH. Although it cannot definitively confirm PH, echocardiography offers a probability-based assessment, enabling clinicians to stratify risk and identify patients who require further testing. Among the several echocardiographic parameters used to assess right ventricular function, tricuspid annular plane systolic excursion (TAPSE) and pulmonary artery systolic pressure (PASP) are widely accepted and validated indices [7].

TAPSE measures the longitudinal movement of the tricuspid annulus toward the cardiac apex during systole. It is an M-mode measurement obtained from the apical four-chamber view and serves as a surrogate for right ventricular systolic function [8]. A TAPSE value >17 mm is considered normal, whereas lower values are indicative of varying degrees of right ventricular dysfunction. As ILD progresses and pulmonary vascular resistance increases due to hypoxia-induced vasoconstriction and remodeling, the right ventricle faces increased afterload, eventually leading to strain and failure. TAPSE, therefore, provides a reliable, reproducible, and readily accessible measure of right heart performance in this context [9].

PASP is estimated non-invasively through Doppler echocardiography by measuring the velocity of the tricuspid regurgitant jet and applying the modified Bernoulli equation: right ventricular systolic pressure (RVSP) = 4 × (TR velocity)² + estimated right atrial pressure. PASP >36 mmHg suggests the presence of PH and is graded as mild (36-49 mmHg), moderate (50-69 mmHg), or severe (≥70 mmHg) [10]. Studies have shown that elevated PASP correlates with increased mortality in ILD patients and reflects the severity of pulmonary vascular remodeling [11]. The combination of reduced TAPSE and elevated PASP has been proposed as a hemodynamic signature for advanced PH and right ventricular dysfunction in ILD.

In addition to echocardiographic markers, functional assessments such as the six-minute walk test (6MWT) and spirometry provide valuable insights into the physiological burden of ILD. The 6MWT is a simple, low-cost, and reproducible submaximal exercise test that reflects a patient’s functional status. The total distance walked in six minutes (6MWD) is influenced by pulmonary, cardiac, and musculoskeletal function and has been validated as a predictor of morbidity and mortality in ILD [12]. The percent predicted 6MWD, calculated using standardized equations accounting for age, sex, height, and weight, enables individualized interpretation of results. Importantly, the degree of oxygen desaturation during the 6MWT serves as a surrogate for gas exchange efficiency. Significant desaturation (≥4% drop in SpO₂ or post-walk SpO₂ <88%) indicates impaired pulmonary function and correlates with disease severity, reduced DLCO, and increased mortality [13]. Such desaturation also correlates with PH and right heart dysfunction, making it a potential early marker for vascular involvement in ILD.

Spirometry remains the mainstay of pulmonary function testing, offering a straightforward, objective readout of lung mechanics. In ILDs, spirometry almost always shows a restrictive pattern: both forced vital capacity (FVC) and forced expiratory volume in one second (FEV₁) are reduced, while the FEV₁/FVC ratio is preserved or even rises. These values are used for tracking disease course and gauging treatment response. FVC, along with the exercise test, has emerged as a robust surrogate for survival in IPF and other fibrotic ILDs [14]. Declining FVC and exercise test performance often parallel rising pulmonary pressures, hinting that spirometry coupled with exercise tests can provide indirect signals of vascular involvement [15].

A broader picture of cardiopulmonary interaction comes from combining three data streams: bedside echocardiography (TAPSE and PASP), the 6WMT (distance covered and exertional desaturation), and routine spirometry (FVC and FEV₁). Prior research has shown that lower TAPSE is associated with shorter walk distances and reduced FVC, while higher PASP is associated with greater oxygen desaturation and spirometry showing a restrictive pattern, associations that carry clinical significance [16]. Yet most of this evidence comes from outside India. We still know little about how often PH complicates ILD in Indian patients, how right heart dysfunction shows up on echocardiography, or how these findings relate to day-to-day functional impairment. Indian patients frequently present later in the disease course, carry heavier comorbidity burdens, and may lack access to advanced diagnostics, factors that underscore the need for local data [17]. Standard reference values for the 6WMT and spirometry are typically borrowed from Western cohorts and may not fit Indian demographics.

Against this backdrop, we designed a prospective study in a South Indian tertiary center to examine how two key echocardiographic measures (TAPSE and PASP) relate to three routine functional assessments, six-minute walk performance, oxygen saturation, and spirometry, in adults with ILD. By clarifying these links, we hope to close an important knowledge gap and boost the clinical usefulness of quick, non-invasive tools for assessing disease burden and guiding follow-up in ILD.

The central objective of this study is to determine whether lower TAPSE and elevated PASP are significantly associated with decreased 6MWD, greater exertional desaturation, and reduced spirometric volumes in ILD patients. We hypothesize that echocardiographic markers of right heart dysfunction and elevated pulmonary pressures are reliable predictors of impaired functional capacity and restrictive ventilatory defects. The goal is to promote an integrated, echocardiography-based approach to early identification of high-risk ILD patients and to provide a foundation for targeted therapeutic interventions. The 6MWD may serve as a functional marker, especially in resource-limited environments.

## Materials and methods

Study design and setting

From March to May 2025, we conducted a prospective, cross-sectional study at Rajarajeswari Medical College & Hospital (RRMCH) in Bengaluru. The Respiratory Medicine team led the work in collaboration with Cardiology. Our goal was to examine how two echocardiographic markers, TAPSE and PASP, correlate with everyday functional measures in ILD. Specifically, we compared these ultrasound readings with six-minute walk distance (6MWD), oxygen saturation (SpO₂), and standard spirometric results.

Ethical approval and consent

The Institutional Ethics Committee at RRMCH approved the protocol (reviewed 28 February 2025, approved 5 March 2025). All procedures complied with the Declaration of Helsinki (2013 revision). Before enrollment, each patient received a detailed explanation of the study’s purpose, procedures, and potential risks, and provided written informed consent. Participant confidentiality was strictly protected: data were coded, anonymized, and used solely for statistical analysis.

Selection criteria

Participants eligible for enrollment were adults (≥18 years) with a definite diagnosis of ILD confirmed by clinical assessment and high-resolution computed tomography (HRCT) of the chest. Diagnostic confirmation strictly followed the joint recommendations of the American Thoracic Society and the European Respiratory Society. Recruitment occurred consecutively from both outpatient clinics and inpatient wards during the study interval.

Exclusion criteria included: (i) any preexisting significant cardiac disorder, including valvular heart disease, previous cardiac surgery, coronary artery disease, clinically relevant arrhythmias, or dependence on a permanent pacemaker; (ii) systemic conditions that could interfere with cardiorespiratory evaluation, such as renal impairment (serum creatinine >1.5 mg/dL), uncontrolled arterial hypertension (blood pressure >180/100 mmHg), or a resting heart rate >120 beats/min; and (iii) musculoskeletal limitations preventing safe completion of the 6MWT.

Echocardiographic evaluation

All participants underwent transthoracic two-dimensional echocardiography performed by a certified cardiologist using the GE Vivid S60N ultrasound system (GE Healthcare, Chicago, Illinois). TAPSE was measured from the apical four-chamber view using M-mode by placing the cursor on the lateral tricuspid annulus and assessing the longitudinal movement during systole. A TAPSE value >17 mm was considered normal, while values between 13-16 mm, 10-12 mm, and <10 mm indicated mild, moderate, and severe right ventricular dysfunction, respectively.

PASP was estimated using the modified Bernoulli equation [18], based on the peak tricuspid regurgitation (TR) jet velocity:

$\text{PASP} = 4\times (\text{TRV})&sup2; + \text{RAP}$

where TRV denotes the peak velocity of the tricuspid regurgitation jet and RAP refers to the estimated right atrial pressure.

RAP was systematically estimated using inferior vena cava (IVC) assessment in the subcostal view according to established guidelines. IVC diameter was measured 2 cm from the right atrial junction during end-expiration, and inspiratory collapse was assessed during spontaneous respiration or the sniff maneuver. RAP values were assigned as follows: IVC diameter <2.1 cm with >50% inspiratory collapse = 3 mmHg; IVC diameter <2.1 cm with <50% inspiratory collapse = 8 mmHg; IVC diameter >2.1 cm with >50% inspiratory collapse = 8 mmHg; and IVC diameter >2.1 cm with <50% inspiratory collapse = 15 mmHg. In cases where IVC assessment was technically difficult, a default RAP of 5 mmHg was used for patients without clinical signs of right heart failure.

The measured PASP values were categorized as normal (<36 mmHg), mildly elevated (36-49 mmHg), moderately elevated (50-70 mmHg), and severely elevated (>70 mmHg).

Six-minute walk test

Functional capacity was evaluated using the 6WMT conducted as per the American Thoracic Society guidelines [19]. The test was administered in a 30-meter flat corridor. Patients were instructed to walk as far as possible at a self-determined pace for six minutes. Continuous monitoring of oxygen saturation and heart rate was done using the Nonin 3150 WristOx2 Pulse Oximeter (Nonin Medical Inc., Plymouth, Minnesota). The distance covered (6MWD) was measured in meters. The predicted 6MWD was calculated using the Enright equation adapted for the Indian population [20]:

$\text{6MWD} = 561.02 &minus; (2.507\times\text{Age in years}) + (1.505\times\text{Weight in kg}) &minus; (0.055\times\text{Height in cm})$

The percentage predicted 6MWD was then determined as

$\frac{\text{Actual 6MWD}}{\text{Predicted 6MWD}}\times100$

Significant desaturation was defined as a drop in SpO₂ of ≥4% from baseline or a final saturation of <88%.

Pulmonary function testing

Spirometric evaluation was undertaken with a handheld MIR Spirobank II device (Medical International Research, Rome, Italy) in line with the 2019 technical recommendations of the European Respiratory Society and the American Thoracic Society. The variables recorded comprised forced vital capacity (FVC), forced expiratory volume in the first second (FEV₁), and the FEV₁/FVC ratio.

Values were reported as percentages of predicted norms using the following validated Indian reference equations:

For males:

$\text{FVC (L)} = 0.06\times\text{Height (cm)} - 0.025\times \text{Age (years)} - 1.76$

$\text{FEV}_{1}\text{ (L)} = 0.048\times\text{Height (cm)} - 0.029\times\text{Age (years)} - 1.06$

For females:

$\text{FVC (L)} = 0.041\times\text{Height (cm)} - 0.018\times \text{Age (years)} - 0.54$

$\text{FEV}_{1}\text{ (L)} = 0.037\times \text{Height (cm)} - 0.022\times \text{Age (years)} - 0.26$

An ethnic correction factor of 0.93 was applied for South Asian populations as per established guidelines. A restrictive ventilatory defect was defined as a reduced FVC with a normal or elevated FEV₁/FVC ratio, an expected pattern in ILD.

Statistical analysis

Data handling and statistical procedures were performed with IBM SPSS Statistics for Windows, Version 27 (Released 2020; IBM Corp., Armonk, New York). Continuous variables are presented as mean ± standard deviation, whereas categorical variables are expressed as frequencies and percentages. Comparisons between two independent groups (e.g., normal vs reduced TAPSE) employed the independent-samples t-test. For comparisons involving three or more categories (e.g., PASP strata), one-way analysis of variance (ANOVA) followed by Bonferroni-adjusted post hoc testing was applied. Pearson correlation coefficients (r) were calculated to explore the relationships between echocardiographic indices (TAPSE, PASP) and functional parameters (6MWD, peripheral oxygen saturation, FVC). A two-tailed p-value <0.05 signified statistical significance.

## Results

**Figure 1:**
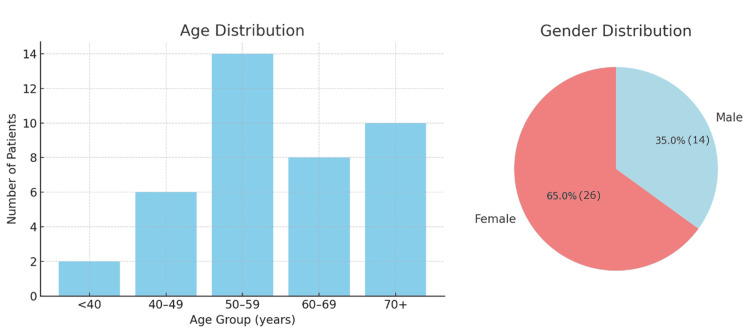
Age and Gender Distribution of Interstitial Lung Disease Patients Demographic characteristics of the study cohort showing age distribution by decades and gender composition. Left panel: Age distribution demonstrates predominance in the 50-69 year age group (n = 22, 55%), followed by 40-49 years (n = 8, 20%), 60-69 years (n = 6, 15%), and ≥70 years (n=4, 10%). Right panel: Gender distribution shows female predominance (n = 26, 65%) compared to males (n=14, 35%). Data represent absolute numbers (n) and percentages (%) of the total study population. Mean age was 58.2 ± 9.6 years.

A total of 40 patients diagnosed with ILD were included in the study. The mean age of the cohort was 58.2 ± 9.6 years, with the majority (55%) falling within the 50- to 69-year age group. Female participants represented a greater proportion of the study population (65%), while males constituted the remaining 35%. The age and gender distribution are shown in Figure 1.

Based on transthoracic echocardiography, 50% of the patients had normal TAPSE values (≥17 mm), indicating preserved right ventricular (RV) systolic function. The remaining 50% had mild RV dysfunction, reflected by TAPSE values between 13 and 16 mm. No cases of moderate or severe TAPSE impairment were recorded. In terms of PASP, 42.5% of patients had normal PASP (<36 mmHg), 50% showed mild elevation (36-49 mmHg), and 7.5% exhibited moderate elevation (50-70 mmHg). This distribution of TAPSE and PASP values is summarized in Table 1.

**Table 1:** Distribution of Echocardiographic Parameters (TAPSE and PASP) Transthoracic 2D echocardiographic findings showing right ventricular function and pulmonary artery pressure distribution in 40 ILD patients. TAPSE (Tricuspid Annular Plane Systolic Excursion) was measured using M-mode from the apical four-chamber view, with normal values defined as ≥17 mm and mild dysfunction as 13-16 mm. PASP (Pulmonary Artery Systolic Pressure) was calculated using the modified Bernoulli equation from tricuspid regurgitation velocity, with normal values <36 mmHg, mild elevation 36-49 mmHg, and moderate elevation 50-70 mmHg. No patients showed severe TAPSE impairment (<13 mm) or severe PASP elevation (>70 mmHg). Data presented as absolute numbers and percentages of total cohort.

Parameter	Number of Patients (n = 40)	Percentage (%)
TAPSE (≥17 mm)	20	50.0%
TAPSE (13–16 mm)	20	50.0%
PASP (<36 mmHg)	17	42.5%
PASP (36–49 mmHg)	20	50.0%
PASP (50–70 mmHg)	3	7.5%

**Figure 2:**
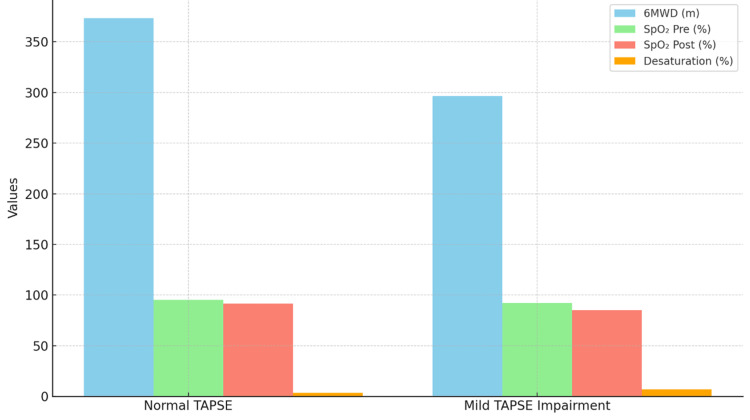
Correlation of TAPSE With 6MWD and Oxygen Saturation Parameters Functional capacity and oxygen saturation comparison between ILD (interstitial lung disease) patients stratified by TAPSE (tricuspid annular plane systolic excursion) values. The left panel shows six-minute walk distance (6MWD) in meters for normal TAPSE (≥17 mm, n = 20) versus mild TAPSE impairment (13-16 mm, n = 20). The right panel displays peripheral oxygen saturation (SpO₂) measured before and after the 6-minute walk test using a Nonin 3150 WristOx2 pulse oximeter. Error bars represent standard deviation. Statistical analysis performed using an independent-samples t-test with p<0.001 indicating highly significant differences between groups. The normal TAPSE group demonstrated superior exercise capacity and better oxygen saturation maintenance during exertion compared to the mild TAPSE impairment group.

Patients with normal TAPSE achieved a significantly greater 6MWD, with a mean of 373.5 ± 34.2 meters. In contrast, patients with mildly reduced TAPSE values recorded a mean 6MWD of 296.4 ± 28.7 meters. The percent predicted 6MWD also followed a similar pattern, favoring those with normal TAPSE. A statistically significant correlation was found between TAPSE and both actual and predicted 6MWD (p = 0.001), as demonstrated in Figure 2. In terms of oxygen saturation, patients with preserved TAPSE values showed higher pre- and post-walk SpO₂ and lower exertional desaturation. Statistical analysis confirmed a significant association between TAPSE and oxygen saturation parameters before and after the 6MWT (p = 0.001). These results are also included in Figure 2, which illustrates both the distance walked and SpO₂ trends by the TAPSE group.

When correlated with spirometry, patients with normal TAPSE showed higher predicted values of forced vital capacity (FVC) and forced expiratory volume in 1 second (FEV₁). The mean FVC in the normal TAPSE group was significantly higher than in those with mild RV dysfunction. The FEV₁/FVC ratio, however, did not significantly differ between groups (p = 0.44). These spirometric results, along with statistical comparisons between TAPSE categories, are presented in Table 2.

**Table 2:** Correlation of TAPSE With Spirometric Parameters Comparison of pulmonary function parameters between ILD patients with normal versus mildly impaired right ventricular systolic function as assessed by TAPSE. Spirometry was performed using the MIR Spirobank II device according to ATS/ERS 2019 guidelines. Values represent the percentage of predicted normal values adjusted for age, sex, height, and ethnicity using validated Indian reference equations. Statistical comparison performed using an independent-samples t-test. P-values <0.05 are considered statistically significant. Data presented as mean ± standard deviation. Normal TAPSE group: n = 20; Mild TAPSE impairment group: n = 20. ILD: interstitial lung disease; TAPSE: tricuspid annular plane systolic excursion; MIR: medical international research; ATS: American Thoracic Society; ERS: European Respiratory Society; FVC: forced vital capacity; FEV₁: forced expiratory volume in 1 second.

Spirometric Parameter	Normal TAPSE (≥17 mm)	Mild TAPSE (13–16 mm)	p-value
FVC (% predicted)	72.5 ± 6.4	61.2 ± 5.7	0.001
FEV₁ (% predicted)	69.1 ± 5.9	58.3 ± 6.2	0.001
FEV₁/FVC ratio	0.82 ± 0.04	0.83 ± 0.05	0.44

**Figure 3:**
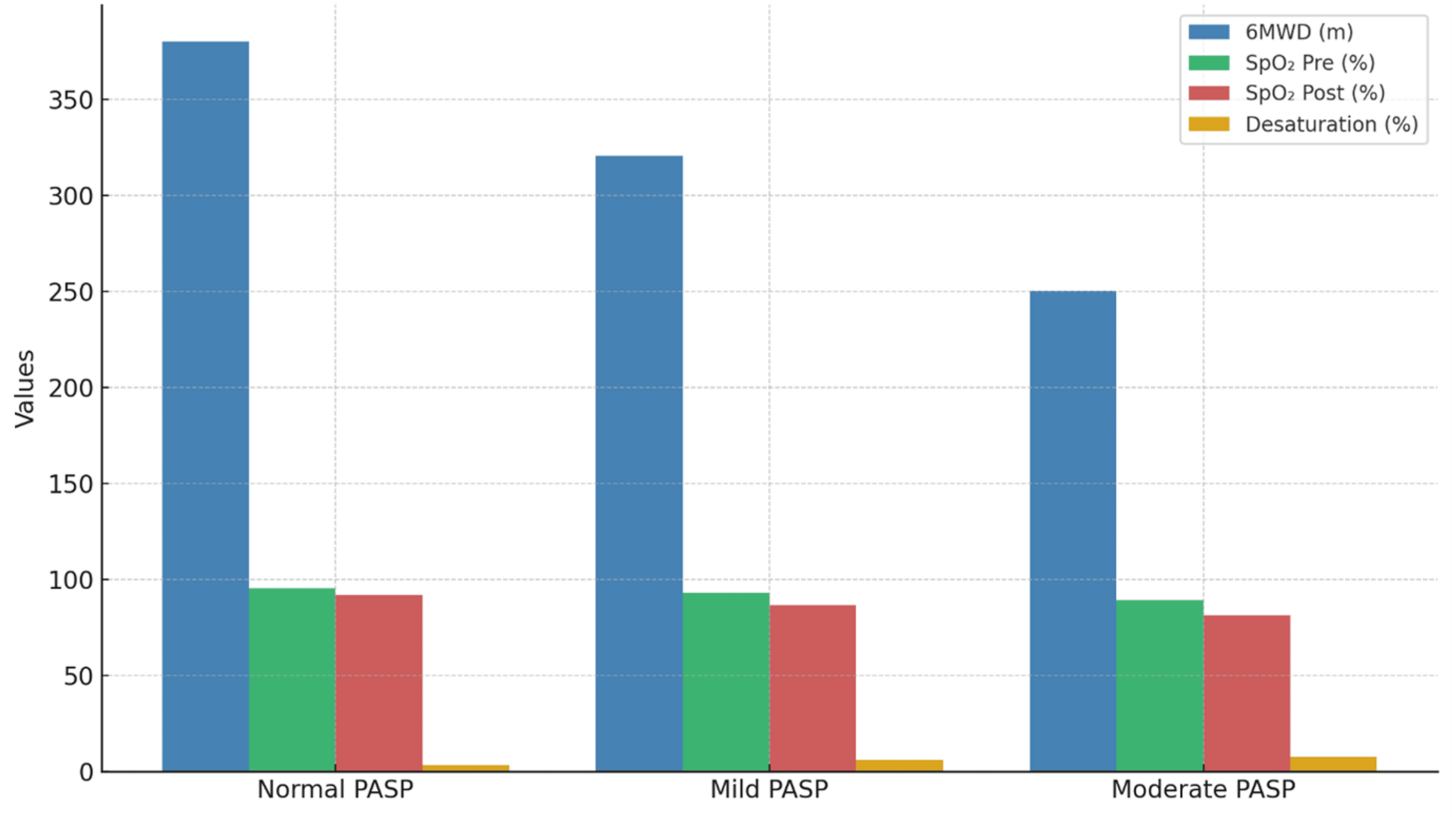
Functional Parameters Stratified by PASP Severity Comprehensive functional assessment across PASP severity groups in ILD patients. The left panel shows 6MWD decline with increasing PASP severity: normal PASP <36 mmHg (n=17), mild elevation 36-49 mmHg (n=20), and moderate elevation 50-70 mmHg (n=3). The right panel demonstrates oxygen saturation patterns before and after the 6-minute walk test across PASP groups, showing progressive desaturation with higher pulmonary pressures. Statistical analysis performed using one-way ANOVA followed by Bonferroni-adjusted post-hoc testing. Error bars represent standard deviation. P<0.001 indicates highly significant differences between groups, with post-hoc analysis revealing significant pairwise differences between all PASP categories for both 6MWD and oxygen saturation parameters. PASP: pulmonary artery systolic pressure; ILD: interstitial lung disease; 6MWD: six-minute walk distance; ANOVA: analysis of variance.

A progressive decline in overall functional capacity was noted with increasing PASP severity. Patients in the normal PASP group consistently outperformed those with mild and moderate PASP elevations across all measured parameters: SpO₂ (pre- and post-6MWT), 6MWD, and spirometric indices. The difference in SpO₂ was statistically significant between PASP groups (p = 0.001), and post hoc analysis showed meaningful differences between all pairwise comparisons. Desaturation increased with PASP severity. These trends are visualized in Figure 3, which combines SpO₂ decline and 6MWD variation across PASP categories.

## Discussion

This prospective study investigated the correlation between two key echocardiographic indicators, TAPSE and PASP, and functional impairment parameters such as 6MWD, oxygen saturation, and spirometric variables in patients with ILD. The findings strongly support the hypothesis that right ventricular dysfunction and PH, as evaluated by non-invasive echocardiography, are significantly associated with reduced functional capacity and greater oxygen desaturation.

The observation that 50% of patients had mild TAPSE impairment and over half showed elevated PASP values underscores the high prevalence of subclinical right heart strain and PH in ILD, even in early stages. This is consistent with earlier studies, which have highlighted that even mild reductions in TAPSE may signal early right ventricular dysfunction and correlate with worsening clinical outcomes in fibrotic lung diseases [21]. TAPSE serves as a robust, reproducible echocardiographic surrogate for right ventricular systolic function and has been validated as a prognostic marker in various forms of ILD [22]. The current study’s finding that patients with lower TAPSE had significantly reduced 6MWD and greater desaturation aligns with prior research, which demonstrated that right heart impairment significantly limits exercise tolerance and promotes exertional hypoxemia [23]. The 6MWT is a widely accepted measure of functional status and has proven predictive value for survival in ILD, particularly in those with fibrotic subtypes [24]. Our study reaffirms this utility by showing clear performance deterioration in patients with reduced TAPSE.

A similar trend was observed with PASP. Patients in the moderate PASP group had poorer oxygen saturation levels both pre- and post-exertion and exhibited greater degrees of desaturation. These trends are corroborated by earlier findings, which indicate that PASP elevation is a significant determinant of exertional limitation in ILD and may reflect progressive pulmonary vascular remodeling [25]. Moreover, the parallel decline seen in spirometric parameters, such as forced vital capacity (FVC) and forced expiratory volume in 1 second (FEV₁), supports the integrated nature of parenchymal and vascular pathology in ILD [26]. One of the strengths of this study lies in the combination of echocardiographic, functional, and spirometric assessment, which offers a more holistic evaluation of disease burden. Earlier literature has often focused on one domain in isolation, but there is growing consensus that such multi-domain analysis better reflects the real-world progression of ILD and aids in personalized treatment planning [27].

Although FEV₁/FVC ratios did not differ significantly between groups, the markedly lower predicted FEV₁ and FVC values in patients with elevated PASP and reduced TAPSE emphasize the restrictive nature of pulmonary dysfunction in ILD. This is in agreement with longitudinal studies that have shown that spirometric decline often parallels increasing pulmonary artery pressure and right heart strain [28]. The observed inverse correlation between PASP and 6MWD further reinforces the clinical impact of vascular involvement on exercise performance, a link also reported in prior echocardiography-based cohorts [29]. Importantly, this study provides clinically relevant evidence from an Indian population, where literature on the integrated echocardiographic and functional assessment in ILD remains limited. Our results indicate that 2D echocardiographic evaluation, when combined with simple bedside tests like 6MWT and spirometry, can offer substantial prognostic value, especially in resource-constrained settings. This aligns with recent recommendations to adopt risk stratification tools tailored to local healthcare capabilities [30].

This study has some limitations that should be acknowledged. Firstly, the sample size was relatively small (n = 40), which may limit the generalizability of the findings. Larger multicenter studies are needed to confirm these associations. Secondly, histopathological subtyping of ILD (e.g., UIP vs. NSIP) was not performed due to a lack of biopsy data, which may have implications for disease trajectory interpretation. Thirdly, follow-up echocardiography and longitudinal outcome tracking were beyond the scope of this study. Additionally, the reliance on echocardiographic estimation of PASP, while clinically practical, may be less accurate than invasive hemodynamic assessment. However, 2D echo remains the most accessible and widely used screening tool in clinical settings, especially where right heart catheterization is not feasible.

The results of this prospective study demonstrate that both TAPSE and PASP derived from 2D echocardiography are significantly associated with functional limitations in ILD patients, as measured by 6MWD, SpO₂ response, and spirometric indices. These findings support the clinical value of integrating echocardiographic assessments with routine functional tests in the comprehensive evaluation of ILD. Right ventricular function and pulmonary artery pressures are not merely secondary markers but play a critical role in determining symptom burden and physical performance. Incorporating such measures may allow for earlier identification of high-risk patients and timely therapeutic intervention. In resource-limited settings, however, 6MWD alone can provide useful insight into the development of PH.

## Conclusions

This study concludes that echocardiographic parameters, specifically reduced TAPSE and elevated PASP, are significantly associated with diminished functional capacity, greater oxygen desaturation, and impaired pulmonary function in patients with ILD. These non-invasive cardiac markers correlate well with key clinical outcomes such as 6MWD and spirometric variables, underscoring their utility in routine assessment and follow-up. Incorporating echocardiography into the multidisciplinary evaluation of ILD can aid in the early detection of PH and right heart dysfunction, even before overt clinical symptoms arise. In resource-constrained settings where 2D echocardiography is not available, the 6MWT can be used as an alternative to assess the development of PH. Future research should focus on larger, multicenter cohorts with long-term follow-up to validate these associations and explore the impact of targeted interventions on right heart function and overall disease progression in ILD.
